# Improvement of Environment and Mechanical Behaviour of Filling Material of Phosphate Solid Waste Using Natural Fibre

**DOI:** 10.3390/ma18173978

**Published:** 2025-08-25

**Authors:** Defeng Liu, Chenglin Ke, Fan Wu, Yantao Zheng

**Affiliations:** 1 School of Resource and Safety Engineering, Wuhan Institute of Technology, Wuhan 430073, China; liudf-whgc@wit.edu.cn (D.L.); 18271574559@163.com (C.K.); 2 Guizhou Xifeng Phosphate Mine Co., Ltd., Guiyang 550003, China; zyt2021best@163.com; 3 Key Laboratory of Mountain Hazards and Engineering Resilience, Institute of Mountain Hazards and Environment, Chinese Academy of Sciences, Chengdu 610213, China; 4 College of Water Resource & Hydropower, Sichuan University, Chengdu 610065, China

**Keywords:** filling material, natural fibre, flowability, mechanical properties, microstructure characterization, porosity

## Abstract

To enhance both the environmental performance and mechanical properties of phosphate solid waste backfill materials, this study examines the effects of corn straw fibre (CS), rice straw fibre (RS), and jute fibre (JF), each at five lengths (3–15 mm) and five dosages (0.1–0.5 wt%), on the rheological behaviour, mechanical strength, and microstructural characteristics of the backfill slurry. The experimental results showed that the incorporation of natural fibres markedly improved both the compressive and tensile strengths of backfill materials. For example, incorporating CS at a length of 12 mm and a dosage of 0.2 wt% increased the compressive and tensile strengths by 144.4% and 18.8%, respectively. Likewise, RS at 3 mm and 0.2 wt% increased the strengths by 68.3% and 11.9%, while JF at 12 mm and 0.5 wt% enhanced them by 108.2% and 14.9%, respectively. Ion leaching experiments and XPS analyses confirmed that the incorporation of natural fibres effectively adsorbed and immobilized phosphorus and fluorine in phosphogypsum. Scanning electron microscopy (SEM) and X-ray diffraction (XRD) analyses revealed that the improved mechanical strength was primarily attributed to fibre-bridging effects and enhanced fibre–matrix bonding. Furthermore, nuclear magnetic resonance (NMR) analysis demonstrated that incorporating natural fibres reduced the porosity of backfill materials (from 12.9% to 8.14%) while increasing their density. This study provides an experimental foundation for optimizing backfill materials and recommends a 12 mm CS fibre length at a dosage of 0.2 wt% to improve the stability and safety of mine fill structures.

## 1. Introduction

China possesses abundant phosphate rock resources, with reserves of 1.9 billion tons in 2022, ranking second globally [1]. Extensive mining and beneficiation in the phosphate rock industry have resulted in substantial by-products, including phosphogypsum, flotation tailings, and gravity tailings. By 2024, the stockpile of phosphorus tailings in China had exceeded 600 million tons, with nearly 10 million tons added each year [2,3]. At present, the predominant method of managing these tailings is to stockpile them near phosphate production sites. However, this practice not only occupies valuable land resources but also poses serious environmental risks, such as groundwater contamination and air pollution [4].

To recycle phosphate mine waste, phosphogypsum is utilized in the chemical, agricultural, and construction sectors. Specifically, its calcium and sulfur content enable chemical production of calcium sulfate and calcium bicarbonate. In agriculture, the acidic components of phosphogypsum help amend saline–alkali soils by adjusting pH. In the construction industry, phosphogypsum can serve as a raw material for producing gypsum blocks and plaster [5], as well as for backfill and cementitious materials [6]. However, the effective utilization of phosphogypsum is severely limited by a low utilization rate (<40%) [7] and high natural radioactivity present in phosphate waste [8,9]. The reuse of flotation and gravity tailings also remains an urgent challenge. Currently, these tailings are primarily employed as backfilling materials in open pits and abandoned mines [10]. Li et al. [11] investigated the use of phosphate tailings as a cementing material for goaf backfilling, examining the influence of different slurry concentrations and ratios on the filling performance. Zhang et al. [12] demonstrated that flotation tailings and gravity tailings, when used as fine aggregates along with conventional cement, significantly enhanced the performance of filling materials. Wang et al. [13] found that adding citric acid and sodium metasilicate to phosphorus tailings enhanced the mechanical properties of the backfill material, reducing its fluidity and preventing premature solidification. However, as mining depths continue to increase, phosphate-based backfill materials fail to meet operational requirements for environmental stability and mechanical strength, particularly in terms of shear and tensile properties, thereby compromising mine safety.

To enhance backfilling materials, various synthetic fibres have been incorporated; however, the high costs associated with synthetic fibres remain a significant barrier, given the large quantities required for each mine goaf. Thus, developing a low-cost filling material to meet the large demand for backfilling materials in goaf areas is crucial. Compared with synthetic fibres, natural fibres are inexpensive and widely available. As an agricultural country, China produces approximately 200 million tons of straw annually [14]. Although straw fibres have been used in construction, livestock breeding, and biogas production, most straw fibres are still disposed of through incineration, causing serious environmental pollution [15,16]. The utilization of natural fibres in backfilling materials not only enhances the performance of the materials but also effectively reduces the environmental impact of phosphate tailings in mining areas. The surfaces of natural fibres contain numerous functional groups, such as hydroxyl and carboxyl groups, which can chemically adsorb or undergo ion exchange with harmful ions, including phosphorus and fluorine. Alkalization of the fibres can further enhance this effect [17,18]. Additionally, the naturally porous structure of the fibres provides numerous adsorption sites, increasing the surface area available for adsorbing harmful ions. A larger specific surface area of the fibres also enhances adsorption efficiency [19]. Furthermore, the environmental friendliness, low cost, and high renewability of natural fibres offer backfilling materials broad application prospects in mine backfilling and environmental restoration.

The application of natural fibres in the field of building materials has been extensively studied, providing a foundation for exploring their use in mine-filling materials. Wang et al. [20] enhanced the mechanical properties of goaf backfill using a mixture of alkalized corn straw and coal gangue. Zhou et al. [21] found that jute fibre improves the flowability and mechanical performance of cement-based backfill materials. Chen et al. [22] demonstrated that adding straw fibre increases the viscosity of the slurry and significantly enhances the compressive strength and elastic modulus of the filling material. Song et al. [23] investigated the dynamic mechanical response and damage evolution of alkaline straw-tailings backfill under cyclic impact loading. Wang et al. [24] examined the effect of rice straw treated with an alkalized solution on the strength of tailings backfill material. Ruan et al. [25] revealed that the intrinsic properties of straw fibre influence the mechanical behaviour of backfill materials. Kirubai et al. [26] developed a silica-filled composite material reinforced with jute fibre and rice straw, and discovered that this material could enhance the tensile elongation and improve the volumetric expansion rate of the samples. Despite the aforementioned studies, research on the effects of fibre type, length, and content on the performance of backfill materials remains limited.

This study investigated the effects of different lengths and dosages of natural fibres on the fluidity and mechanical properties of backfilling materials. Furthermore, variations in the strength and failure patterns of the materials were analysed using uniaxial compressive and tensile strength tests. The influence of natural fibres on the microstructure and composition of the materials was examined through scanning electron microscopy (SEM), X-ray diffraction (XRD), and nuclear magnetic resonance (NMR) analysis. The environmental impact of backfill materials and the adsorption mechanisms of ions by natural fibres were evaluated using ion chromatography (IC) and X-ray photoelectron spectroscopy (XPS). The findings provide a basis for the application of natural fibres in mine backfill materials to enhance stability and safety in goaf areas.

## 2. Materials and Methods

### 2.1. Materials

#### 2.1.1. Aggregate

**Figure 1:**
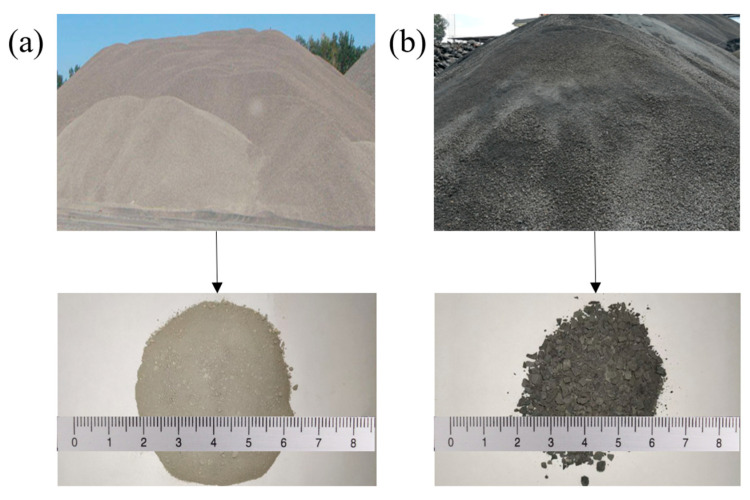
(**a**) Flotation tailings and (**b**) gravity tailings.

In this study, phosphate mine tailings from a local mining company in Yichang, Hubei Province, China, were used as filling aggregates. The tailings consisted of gravity and flotation tailings, both of which were alkaline (Figure 1). Flotation tailings, with a moisture content of 8% and particle sizes ranging from 109 µm to 0.338 mm, were used as fine aggregates. Gravity tailings with a moisture content of 3% and particle sizes ranging from 199 µm to 4.26 mm, were employed as coarse aggregates. Table 1 lists their chemical compositions: flotation tailings primarily contain CaO and SiO_2_, while gravity tailings consist mainly of CaO, SiO_2_, and P_2_O_5_.

#### 2.1.2. Binder

**Figure 2:**
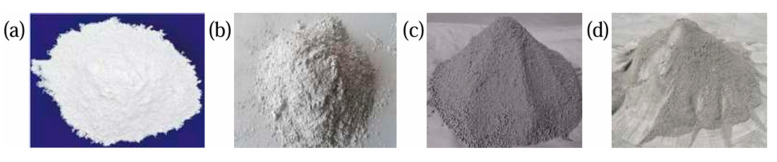
(**a**) Quicklime; (**b**) slag powder; (**c**) phosphogypsum; (**d**) cement.

The binder consisted of P.O42.5 Portland cement, S95 grade slag, and lime-modified phosphogypsum (Figure 2). Commercial P.O42.5 Portland cement and S95 grade slag were sourced locally, while raw phosphogypsum was obtained from a phosphate mining company. The phosphogypsum exhibited a powdery morphology with a moisture content of 13%. Table 2 presents their chemical compositions: phosphogypsum required alkaline treatment due to its acidity, whereas slag primarily contained CaO and SiO_2_. Based on previous studies [20,27], the cement phases included C_3_S, C_2_S, C_3_A, and C_4_AF.

#### 2.1.3. Natural Fibres

**Figure 3:**
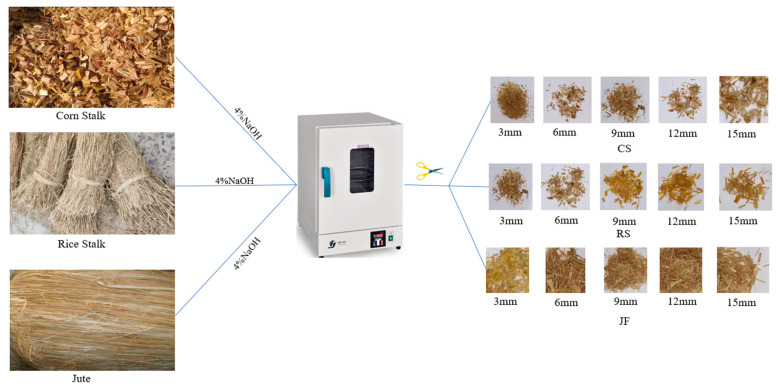
Schematic diagram of natural fibre treatment.

**Figure 4:**
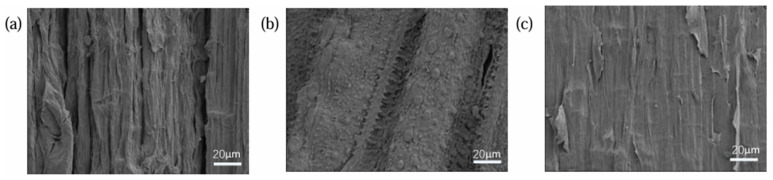
Microscopic images of natural fibres ((**a**) CS, (**b**) RS, (**c**) JF).

As shown in Figure 3, three types of natural fibres—CS, RS, and JF—were used in this study. Figure 4 presents microscopic images of these fibres. The surface of CS exhibits a rugged morphology with deep grooves, while RS displays a distinct wavy pattern covered with protrusions. In contrast, the surface of JF is relatively smooth but contains numerous small fibrillar structures.

As indicated in Table 3, these fibres are primarily composed of cellulose, hemicellulose, lignin, and ash [28,29,30]. Cellulose and hemicellulose are polysaccharides that hydrolyse into simple sugars under alkaline conditions, which may inhibit cement hydration [31]. To mitigate this effect, all natural fibres were soaked in a 4 wt% NaOH solution for 24 h, rinsed to a pH of 7.0 ± 0.1, and oven-dried at 50 °C for 6 h before use [32,33,34]. Fibre lengths of 3, 6, 9, 12, and 15 mm were employed to investigate the length-dependent effects on backfill performance.

### 2.2. Sample Preparation

Based on our preliminary experimental results, the optimal solid content was set at 76%, with a mix ratio of cement: phosphogypsum: slag: gravity separation tailings: flotation tailings: water = 1.05:0.34:0.15:3.64:2.64:2.15. Quicklime (2 wt%) was incorporated into the phosphogypsum for modification. The mix proportions are shown in Table 4, with CS, RS, and JF representing corn stalk fibre, rice stalk fibre, and jute fibre, respectively. The experiment was conducted in two stages, examining the effects of fibre length (RS1–5, CS1–5, and JF1–5) and fibre content (RS6–9, CS6–9, and JF6–9). In the first stage, the effects of fibre length (3, 6, 9, 12, and 15 mm) were studied at a fixed fibre content of 0.1 wt%. In the second stage, the effects of natural fibre content (0.1, 0.2, 0.3, 0.4, and 0.5 wt% by weight of the total filling aggregate) on backfill materials were investigated using the optimal fibre length.

Samples were prepared by first mixing the cementing material with the filling aggregates, followed by the addition and thorough mixing of the natural fibres. The slurry was used for flowability testing, and the remaining slurry was poured into molds. After 24 h of setting, the solidified specimens were demolded and placed in a curing chamber at 25 ± 1 °C with 95% relative humidity. Curing ages were designated as 7, 14, and 28 days before mechanical property testing.

### 2.3. Test Methods

**Figure 5:**
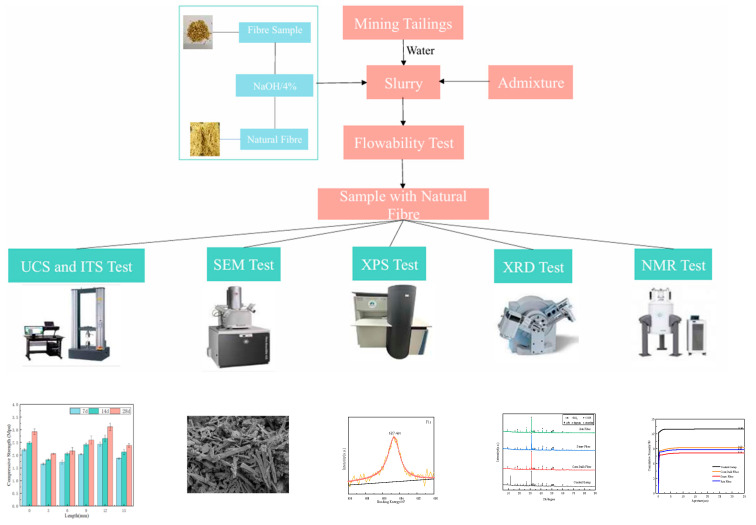
Schematic diagram of all tests in this study.

This study evaluated the flowability, mechanical strength, microstructure, crystal composition, and porosity of all specimens. Standard specimen dimensions were as follows: 70.7 mm cubes for compressive strength testing, 100 mm cubes for tensile strength testing, and Ø50 mm × 100 mm cylinders for nuclear magnetic resonance (NMR) analysis [35]. All results represent the average of at least three replicates. Figure 5 provides a schematic overview of the experimental procedures.

#### 2.3.1. Flowability Test

The flowability of all specimens was evaluated through slump and setting time tests in accordance with GB/T 50080-2016 [36]. Slump was measured using a standard slump cone (top Ø = 100 mm, base Ø = 200 mm, height = 300 mm), and setting times were recorded in minutes.

#### 2.3.2. Mechanical Strength

Tensile and compressive strengths were measured using a microcomputer-controlled electro-hydraulic servo pressure tester by GB/T 23561.12-2010 [37], at a loading rate of 1200 N/min. Experimental data were recorded at 0.5 s intervals. Tensile strength tests followed the same loading procedure. Three specimens per backfill ratio were evaluated, and compressive and tensile strength results were reported as averages.

#### 2.3.3. SEM Analysis

Samples cured for 28 days underwent scanning electron microscope (SEM) analysis. First, specimens were soaked in anhydrous ethanol for 24 h to terminate hydration, and then oven-dried at 50 °C. The dried samples were sputter-coated with gold to enhance conductivity for SEM imaging.

#### 2.3.4. XRD Analysis

X-ray diffraction (XRD) analysis was performed on specimens cured for 28 days to characterize hydration products and their crystallographic intensities, using a scanning range of 5° to 90° at a rate of 5°/min.

#### 2.3.5. Porosity Test

The porosity of specimens cured for 28 days was determined using nuclear magnetic resonance (NMR) analysis. This technique utilizes hydrogen nuclei signals within magnetic fields to characterize pore structures, deriving pore parameters through spatial mapping of water molecules.

#### 2.3.6. Ion Test

The concentration of phosphorus (P) and fluorine (F) in sample leachates was determined using ion chromatography (IC) to evaluate the environmental impact of backfill materials. Adsorption mechanisms of these ions by natural fibres were investigated via X-ray photoelectron spectroscopy (XPS).

## 3. Results and Discussion

### 3.1. Flowability

#### 3.1.1. Slump

**Figure 6:**
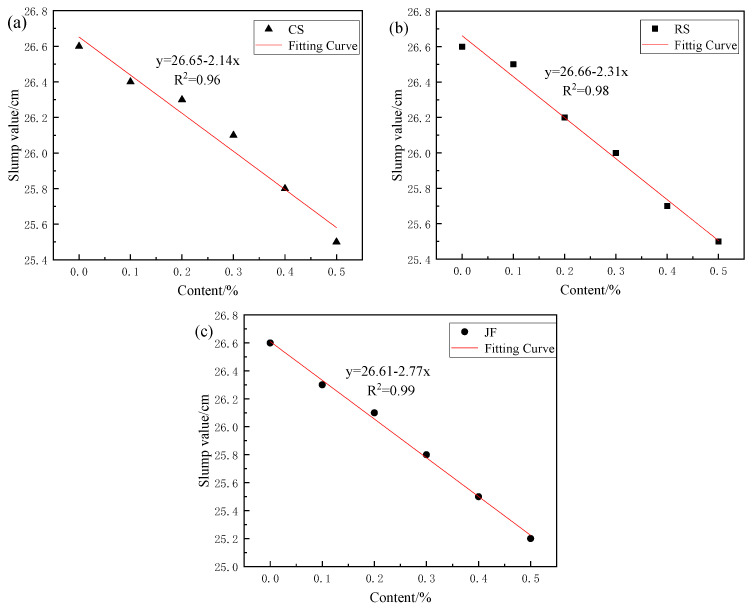
Slump of slurry with natural fibres. (**a**) CS, (**b**) RS, (**c**) JF.

As shown in Figure 6, the slump of the Ref. and slurries with natural fibres varied with increasing fibre content. As the natural fibre content increased from 0 wt% to 0.5 wt%, the slump of slurries with CS, RS, and JF decreased from 26.6 cm to 25.5 cm, 25.5 cm, and 25.2 cm, respectively, representing decreases of 4.1%, 4.1% and 5.3%. This indicates a slight decreasing trend, exhibiting a strong linear relationship; with the addition of JF showing the strongest linear correlation. This trend in slump for slurries with natural fibres is consistent with that reported in the literature.

#### 3.1.2. Setting Time

**Figure 7:**
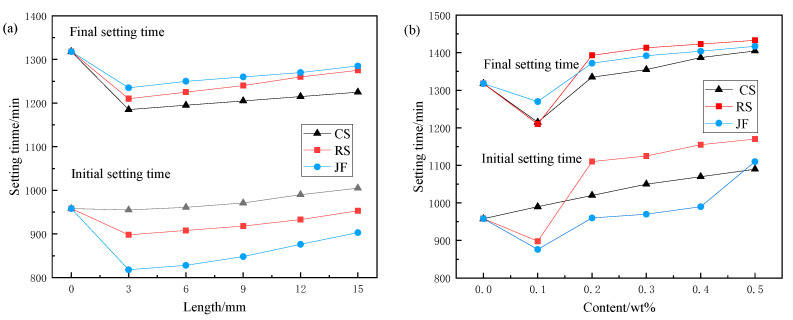
Setting time of slurry with natural fibres. (**a**) Fibre length, (**b**) Fibre content.

As shown in Figure 7, increased fibre length resulted in longer setting times, with more pronounced delays in initial setting than in final setting. The final setting consistently occurred earlier than that of the Ref., indicating that fibre length primarily affected the initial setting. When fibre content increased from 0.2 wt% to 0.5 wt%, setting times exceeded Ref. values, particularly for RS, which exhibited the longest durations: 22.1% and 4.9% increases in initial and final setting times, respectively. These transitions from fluid to solid define the critical construction window for effective filling operations.

The effects of different natural fibre types and contents on slurry flowability are likely due to their high water absorption, which reduces the free water in the slurry. Each fibre type affects flowability differently based on its distinct water absorption rate [38,39]. Furthermore, variations in fibre morphology and properties, including fibre length, diameter, and surface characteristics (Figure 4), increase slurry viscosity and affect flowability [40,41]. In addition, the surface substances and hydrophilic functional groups of natural fibres interact with the slurry, resulting in physical adsorption and chemical bonding that further influence flowability [42]. The uniform dispersion of natural fibres in the slurry also affects viscosity and overall fluidity [43].

### 3.2. Porosity

**Figure 8:**
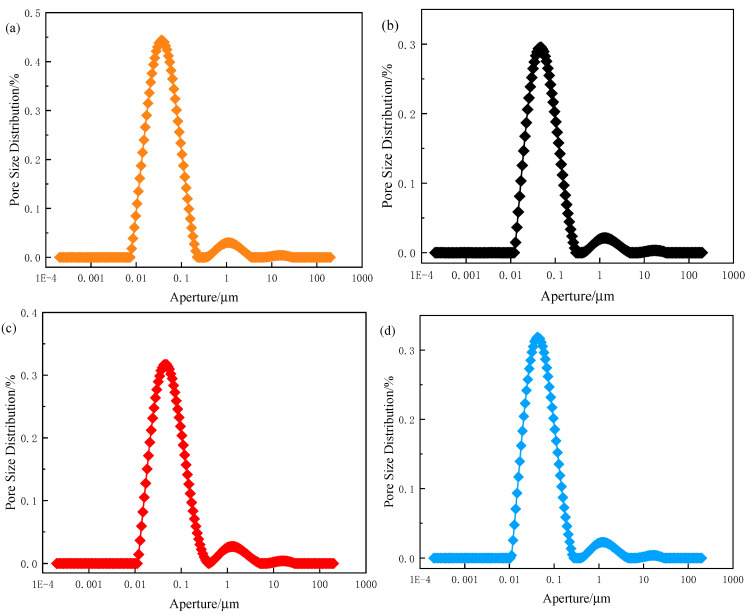
Pore distribution of backfill material ((**a**) Ref., (**b**) CS, (**c**) RS, (**d**) JF).

**Figure 9:**
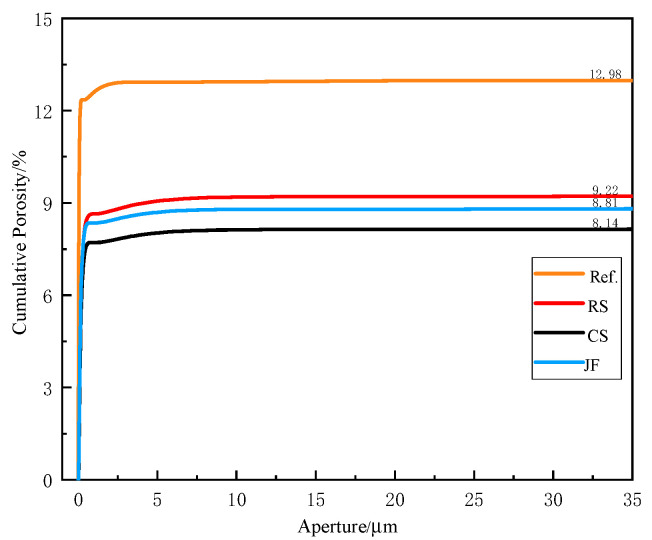
Cumulative porosity of backfill material.

Pore distribution and cumulative porosity results for the sample with the highest compressive strength among the three natural fibre types at 28 days are presented in Figure 8 and Figure 9, respectively. The Ref. sample exhibited 12.9% porosity, while CS, RS, and JF-incorporated samples showed porosities of 9.2%, 8.1%, and 8.8%, corresponding to reductions of 28.9%, 37.2%, and 32.1%, respectively, compared to the Ref. The addition of natural fibres significantly reduced porosity across all specimens. For pore sizes predominantly within the 0–1 μm range, porosity increased linearly with the size range, indicating a dominant micropore content [44].

### 3.3. XRD

**Figure 10:**
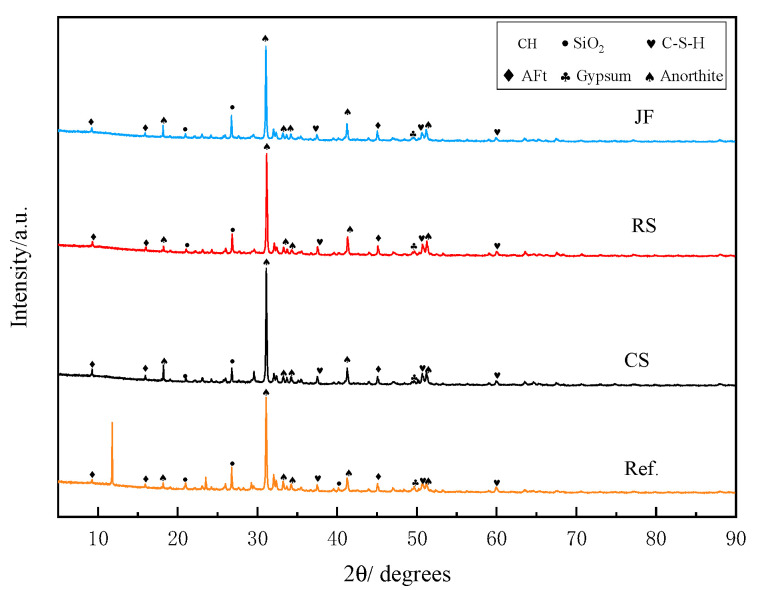
X-ray diffraction pattern of sample with or without natural fibre.

XRD analysis results for the sample with the highest compressive strength among the three natural fibre types at 28 days are presented in Figure 10. Hydration products, including ettringite (Aft), calcium hydroxide (CH), and calcium silicate hydrate (C-S-H), formed during hydration and contributed to the sample’s mechanical strength [45]. No new diffraction peaks were observed with natural fibre incorporation. However, altered intensities of hydration product peaks indicate more complete hydration reactions due to fibre addition. The enhanced formation of hydration products improves the bonding between the natural fibres and the matrix, thereby enhancing the sample’s mechanical strength [22]. The diffraction peaks corresponding to hydration products differ among the fibres. The diffraction peaks of the hydration products vary, with CS showing the strongest peaks, followed by RS and JF [46]. This indicates that the adhesion between CS and the matrix may be stronger than that of the other two fibres, and CS most significantly enhances the compressive strength of the backfill material. These findings align with compressive strength results discussed below.

### 3.4. Compressive Strength

#### 3.4.1. Effect of Fibre Length on Compressive Strength

**Figure 11:**
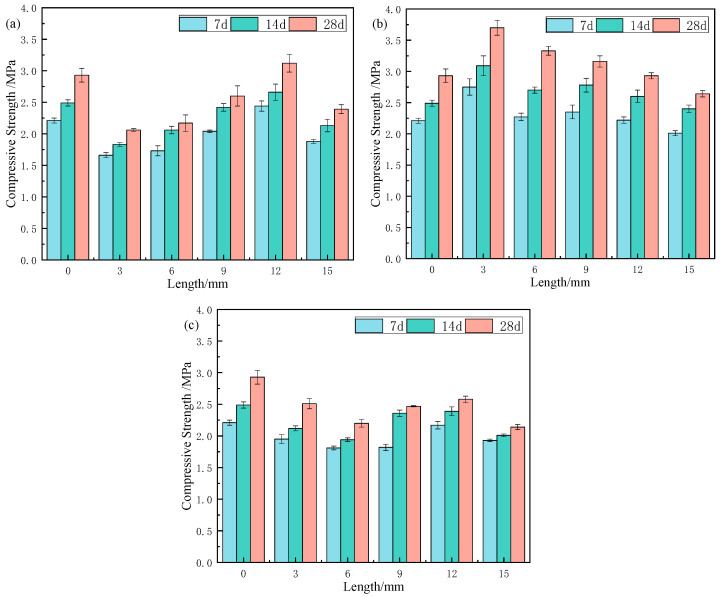
Compressive strength of samples with different natural fibre lengths ((**a**) CS, (**b**) RS, (**c**) JF).

As shown in Figure 11, the compressive strength of samples with CS initially increased as the fibre length increased from 3 mm to 12 mm, peaking at 12 mm (3.12 MPa) before decreasing. For RS, compressive strength decreased with increasing fibre length, with an optimal length of 3 mm (3.70 MPa). For JF, compressive strength followed a “decreasing-increasing-decreasing” trend, with turning points at 3 mm and 12 mm, and the 12 mm JF sample (2.58 MPa) exhibited the highest strength for this fibre type. Overall, CS at 12 mm, RS at 3 mm, and JF at 12 mm provided the most favourable improvements.

Compared to the Ref. at curing ages of 7, 14, and 28 days, samples containing 12 mm CS showed increase of 10.4%, 10.4%, and 6.5%, respectively; samples with 3 mm RS increased by 24.4%, 28.2%, and 26.3%; whereas samples with 12 mm JF decreased by 1.8%, 0.9%, and 12.1%. Comparing fibre types, CS at 12 mm and RS at various lengths (3–12 mm) outperformed the Ref., while JF-containing samples remained lower. These results confirm that different natural fibres exert distinct effects on compressive strength.

**Figure 12:**
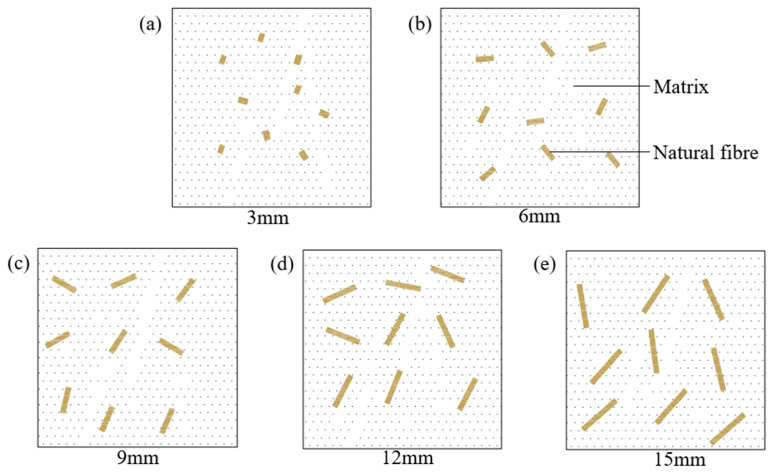
Diagram of different fibre lengths in backfill material. (**a**) 3 mm, (**b**) 6 mm, (**c**) 9 mm, (**d**) 12 mm, (**e**) 15 mm.

Figure 12 illustrates the distribution of natural fibres in the backfill material. When fibres are relatively short, their contact area with the matrix is limited, resulting in defects and weak bonding. Once a fibre reaches a critical length, its entire surface and both ends come into full contact with the matrix, enhancing bond strength and optimizing compressive strength [47]. The sensitivity of samples to fibre length varies by fibre type. Based on compressive strength results (Figure 11), the descending order of sensitivity is RS > CS > JF, likely due to differences in the chemical composition of each fibre type, which influence their properties [48].

Fibre shape also affects performance. CS is blocky, RS is tubular, and JF is filamentous [28,29,30]. The tubular RS provides a large contact area and integrates well with the slurry, maximizing hydration reaction improvement; however, beyond the critical length, the slurry cannot fill the interior, reducing bonding strength. The blocky CS similarly offers a large contact area, which increases with fibre length and peaks at the critical length, maximizing compressive strength [49,50]. In contrast, filamentous JF has a limited contact area due to its lower addition and partially inhibits hydration, resulting in weaker bonding and reduced compressive strength [46].

#### 3.4.2. Effect of Fibre Content on Compressive Strength

**Figure 13:**
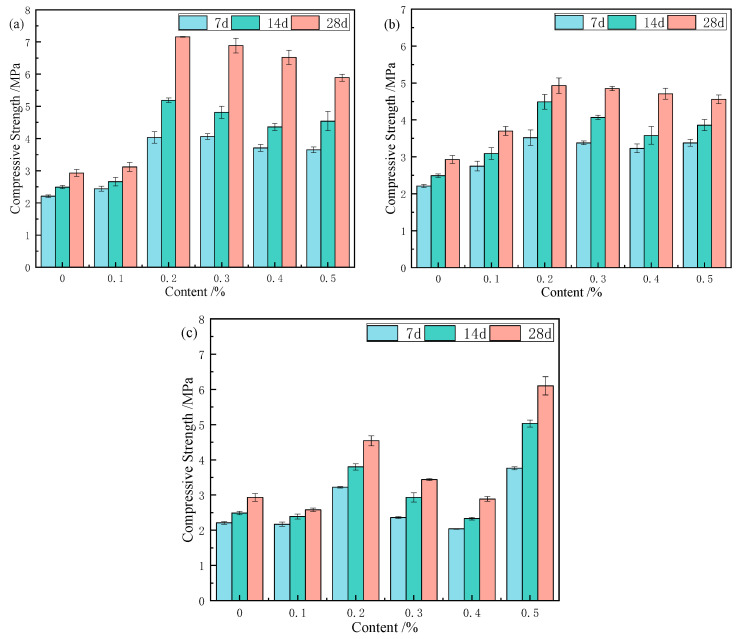
Compressive strength of samples with different fibre contents ((**a**) CS, (**b**) RS, (**c**) JF).

Based on the optimized fibre lengths, 12 mm CS, 3 mm RS, and 12 mm JF were selected to investigate the effects of fibre content on compressive strength, as shown in Figure 13. When fibre content increased from 0.1 wt% to 0.5 wt%, CS and RS samples exhibited similar trends: strength first increased and then decreased, peaking at 0.2 wt%. In contrast, JF samples followed an increase–decrease–increase pattern, reaching peak strength at 0.5 wt%. Therefore, the optimal contents are 0.2 wt% for CS and RS, and 0.5 wt% for JF.

With curing age increasing from 7 to 28 days, compressive strength of 0.2 wt% CS samples increased by 82.4%, 115.4%, and 144.4%, respectively, compared to Ref. Similarly, 0.2 wt% RS samples increased by 24.4%, 28.2%, and 26.3%, while 0.5 wt% JF samples increased by 70.1%, 108.7%, and 108.2% at 7, 14, and 28 days. The results indicate that natural fibre content significantly enhances compressive strength. Improvements with CS, particularly at 0.2 wt%, became more pronounced between 14 and 28 days, surpassing the other fibres. Except for JF, all CS and RS samples (0.1–0.5 wt%) exceeded the Ref. strength. Compared with the mine backfill strength standard reported by [51], samples containing CS (12 mm, 0.2 wt%), RS (3 mm, 0.2 wt%), and JF (12 mm, 0.5 wt%) all met or exceeded the standard, confirming the effectiveness of natural fibres.

**Figure 14:**
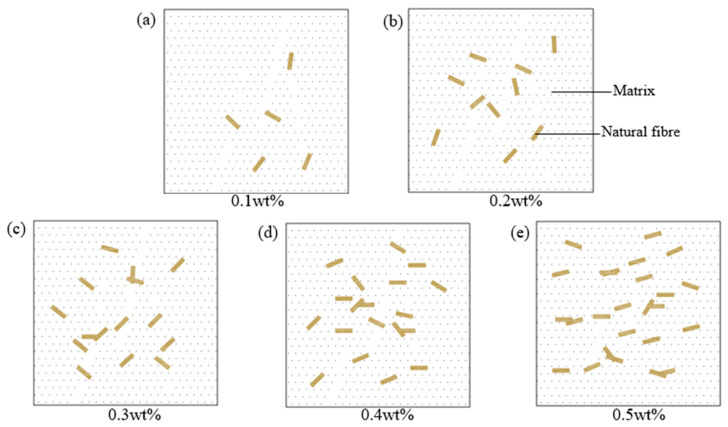
Diagram of different fibre content in backfill material. (**a**) 0.1 wt%, (**b**) 0.2 wt%, (**c**) 0.3 wt%, (**d**) 0.4 wt%, (**e**) 0.5wt%.

Figure 14 illustrates the natural fibre distribution within the matrix, highlighting bridging effects [52]. Increasing fibre content expands the stress-bearing area, strengthens fibre-matrix bonding, inhibits crack propagation, improves detachment resistance, and enhances compressive strength [53]. However, alkalized fibres still inhibit hydration reactions, and higher content intensifies this inhibition [54], reducing fibre-matrix bond strength. Below the optimal content, bridging effects dominate, leading to increased strength [50]. Beyond the critical content, fibre overlapping reduces fibre-matrix contact area, introduces defects, and weakens bonding, resulting in reduced compressive strength [55]. Fibre morphology also influences content effects [56]. At 0.5 wt% JF, the maximal strength indicates optimal bonding and bridging.

From a porosity perspective, natural fibre addition enhances mechanical properties through several mechanisms: fibres act as internal curing agents. As curing age increases, hydration products progressively cover fibre surfaces, reducing matrix pores and strengthening fibre-matrix bonding [57,58]. The reduction in porosity improves compressive strength [59], decreases microcrack connectivity, and inhibits the formation of stress-concentration paths. This enhances stress transfer under load, further improving mechanical properties. Additionally, lower porosity reduces material permeability and water absorption, increasing safety and stability in goaf areas [46,60,61].

### 3.5. Tensile Strength

#### 3.5.1. Effect of Fibre Length on Tensile Strength

**Figure 15:**
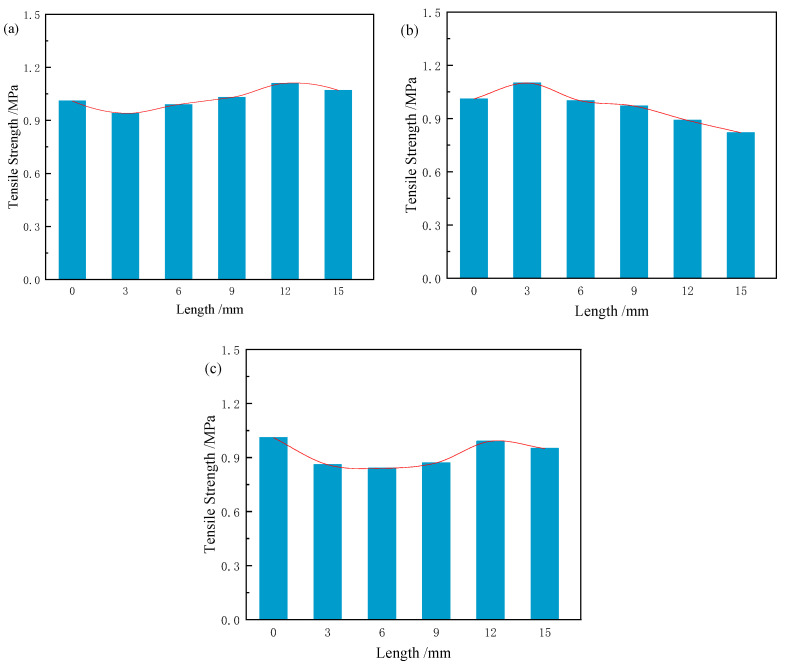
Tensile strength of sample with different fibre lengths ((**a**) CS, (**b**) RS, (**c**) JF).

As shown in Figure 15, when natural fibre length increases from 3 mm to 15 mm, the tensile strength of CS samples first increases and then decreases, with the optimal length being 12 mm. The tensile strength of RS samples shows a consistent decrease, with the optimal length at 3 mm. For JF, tensile strength initially increases and then decreases, peaking at 12 mm. These results indicate that the optimal fibre lengths for improving tensile strength are 12 mm for CS, 3 mm for RS, and 12 mm for JF, with corresponding tensile strengths of 1.11 MPa, 1.10 MPa, and 0.99 MPa, respectively. This trend mirrors the effects observed on compressive strength. Compared to the Ref. (1.01 MPa), the tensile strength of samples with CS (12 mm), RS (3 mm), and JF (12 mm) increased by 5.9% and 8.9% and decreased by 2%, respectively.

**Figure 16:**
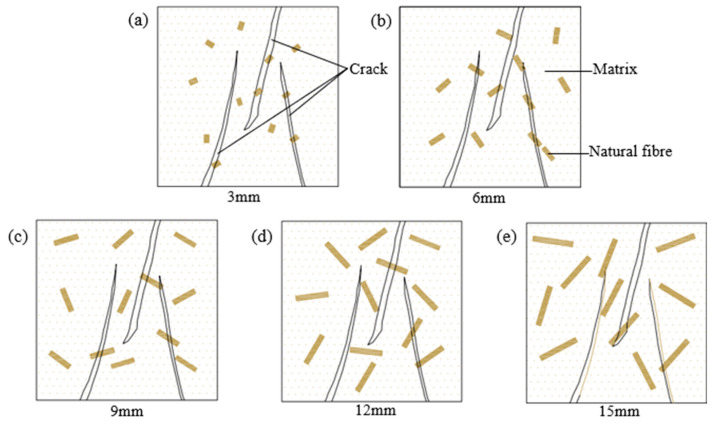
Diagram of different fibre lengths in damaged samples. (**a**) 3 mm, (**b**) 6 mm, (**c**) 9 mm, (**d**) 12 mm, (**e**) 15 mm.

As shown in Figure 16, natural fibres of various lengths are randomly distributed within the matrix. During tensile failure, fibres provide resistance by bridging across the matrix, inhibiting crack propagation. This bridging requires additional energy to pull fibres from the matrix, thereby increasing tensile strength [62]. The effectiveness of bridging depends on fibre position and type. Tensile reinforcement is related to the number of fibres intersecting fracture surfaces. Short tubular RS fibres fill the slurry more completely, reducing cavities and enhancing bonding. When RS fibres are longer, complete filling becomes difficult, explaining the shorter optimal length. For CS and JF, exceeding the optimal fibre length leads to clustering, which likely reduces tensile strength [48].

#### 3.5.2. Effect of Fibre Content on Tensile Strength

**Figure 17:**
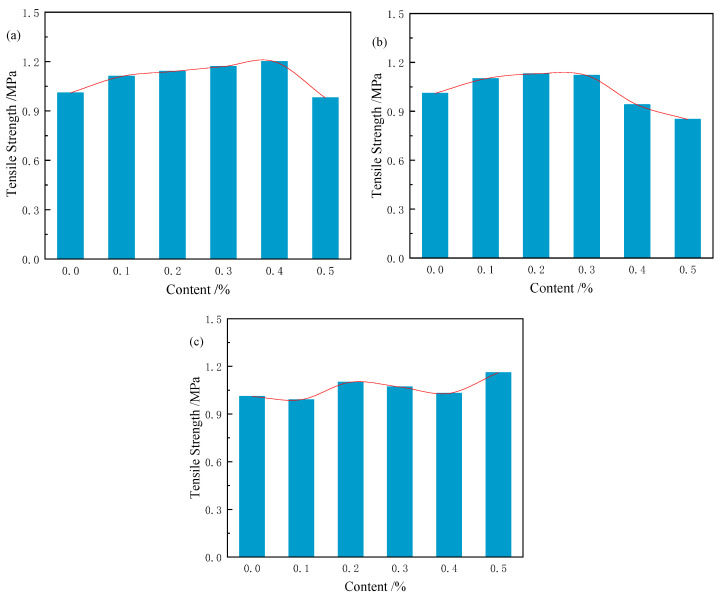
Tensile strength of sample with different fibre content ((**a**) CS, (**b**) RS, (**c**) JF).

As shown in Figure 17, when natural fibre content increases from 0.1 wt% to 0.5 wt%, the tensile strength of samples with CS and RS first increases and then decreases, with optimal contents of 0.4 wt% for CS and 0.2 wt% for RS. For samples with JF, tensile strength reaches a maximum at an optimal content of 0.5 wt%. The corresponding tensile strengths of CS (0.4 wt%), RS (0.2 wt%), and JF (0.5 wt%) are 1.20 MPa, 1.13 MPa, and 1.16 MPa, respectively, representing increases of 18.8%, 11.9%, and 14.9% compared to the Ref.

**Figure 18:**
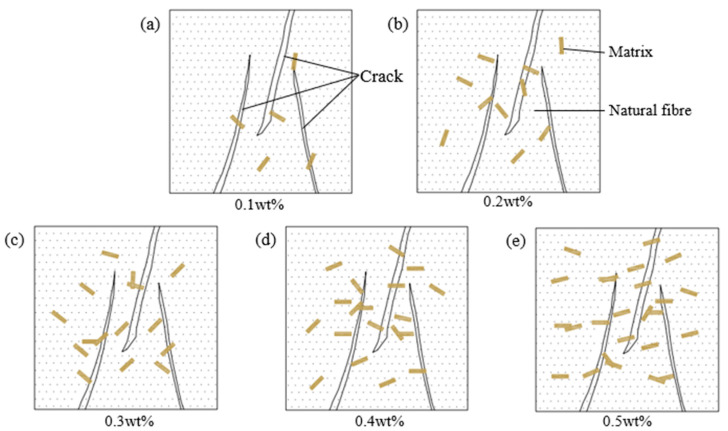
Diagram of different fibre contents in damaged samples. (**a**) 0.1 wt%, (**b**) 0.2 wt%, (**c**) 0.3 wt%, (**d**) 0.4 wt%, (**e**) 0.5 wt%.

As shown in Figure 18, during tensile failure of natural fibre-reinforced samples, fibres spanning the damaged zone act as anchors, bridging cracks and resisting propagation while providing tensile resistance across the fracture plane. Extracting these fibres requires higher energy, thereby enhancing sample strength [63]. Increasing fibre content raises the number of fibres crossing cracks, amplifying crack-arresting capacity and initially improving tensile strength. However, exceeding a critical fibre threshold leads to overlapping and clumping, reducing fibre-matrix contact at the fracture interface. This weakens load-transfer efficiency, causing strength to decrease with further fibre addition [48]. In this study, blocky CS achieves superior crack resistance due to its larger matrix contact area. Tubular RS reaches optimal enhancement at 0.2 wt%, whereas JF maximizes tensile strength at 0.5 wt% by optimizing interfacial engagement at the fracture plane [62]. Compared to previous studies [58,64,65] on mine backfill, these results demonstrate superior tensile strength improvement, validating the efficacy of CS (12 mm, 0.4 wt%), RS (3 mm, 0.2 wt%), and JF (12 mm, 0.5 wt%) for reinforcing phosphate tailings materials.

The mechanical strengths presented in Table 5 are compared with findings from other relevant studies. Variations in mechanical strength arise from differences in fibre type and backfill composition. The three natural fibres used in this study exhibit greater improvements in compressive strength than those reported in the literature. Although tensile strength enhancements vary slightly, these fibres generally improve the tensile performance of backfill materials. Overall, the natural fibres investigated are notable for their availability, low cost, and effectiveness, indicating that their combination with phosphate tailings offers a promising approach for mine backfilling. This highlights the potential applicability of natural fibres across diverse mine types, broadening their utility in the backfill sector.

### 3.6. Failure Mode Analysis of Filling Materials

#### 3.6.1. Macroscopic Failure Mode

**Figure 19:**
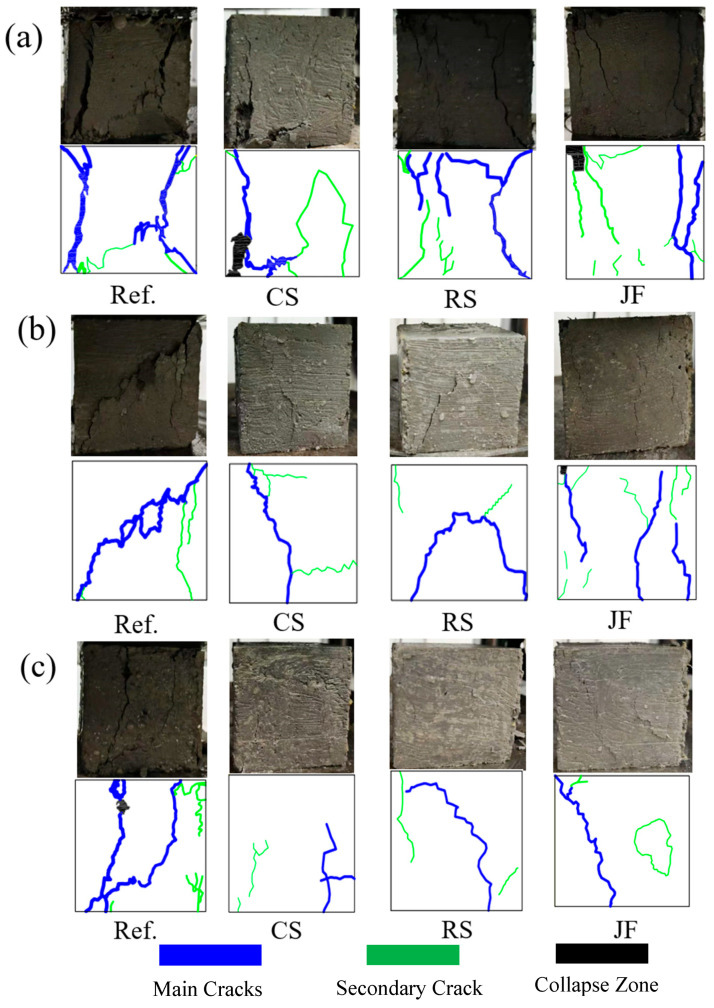
Failure mode diagram of samples with different natural fibres ((**a**) 7d, (**b**) 14d, (**c**) 28d).

As shown in Figure 19, the macroscopic failure morphologies of the Ref. and fibre-reinforced samples differ markedly, indicating that natural fibres influence failure modes. At 7 days, the Ref. sample exhibited primarily tensile failure, with two main lateral cracks nearly splitting the specimen and multiple new cracks forming [63]. Fibre-reinforced samples displayed milder damage, with narrower main cracks and fewer secondary cracks. While tensile failure remained dominant, some shear failure was observed. At 14 and 28 days, Ref. samples continued to show tensile-dominated failure with minor shear, whereas fibre-reinforced samples developed predominantly shear failure, lacking penetrating main cracks. Their narrower cracks preserved structural integrity and load-bearing capacity [68].

The analysis indicates that natural fibres form bonds across damaged sections, enhancing sample integrity [69]. During failure, fibres bridge gaps and exert tensile forces, strengthening the matrix connection. This bonding increases the energy absorbed during failure, thereby improving compressive strength, with effects becoming more pronounced at longer curing times. In this study, CS-reinforced samples exhibited the highest compressive strength and least damage, followed by RS, while JF samples showed the lowest performance [70]. These differences arise from the distinct morphology and properties of each fibre type and align with the mechanical strength results, confirming the effectiveness of natural fibres in enhancing strength and reducing structural damage in backfill materials.

#### 3.6.2. Microscopic Failure Mode

**Figure 20:**
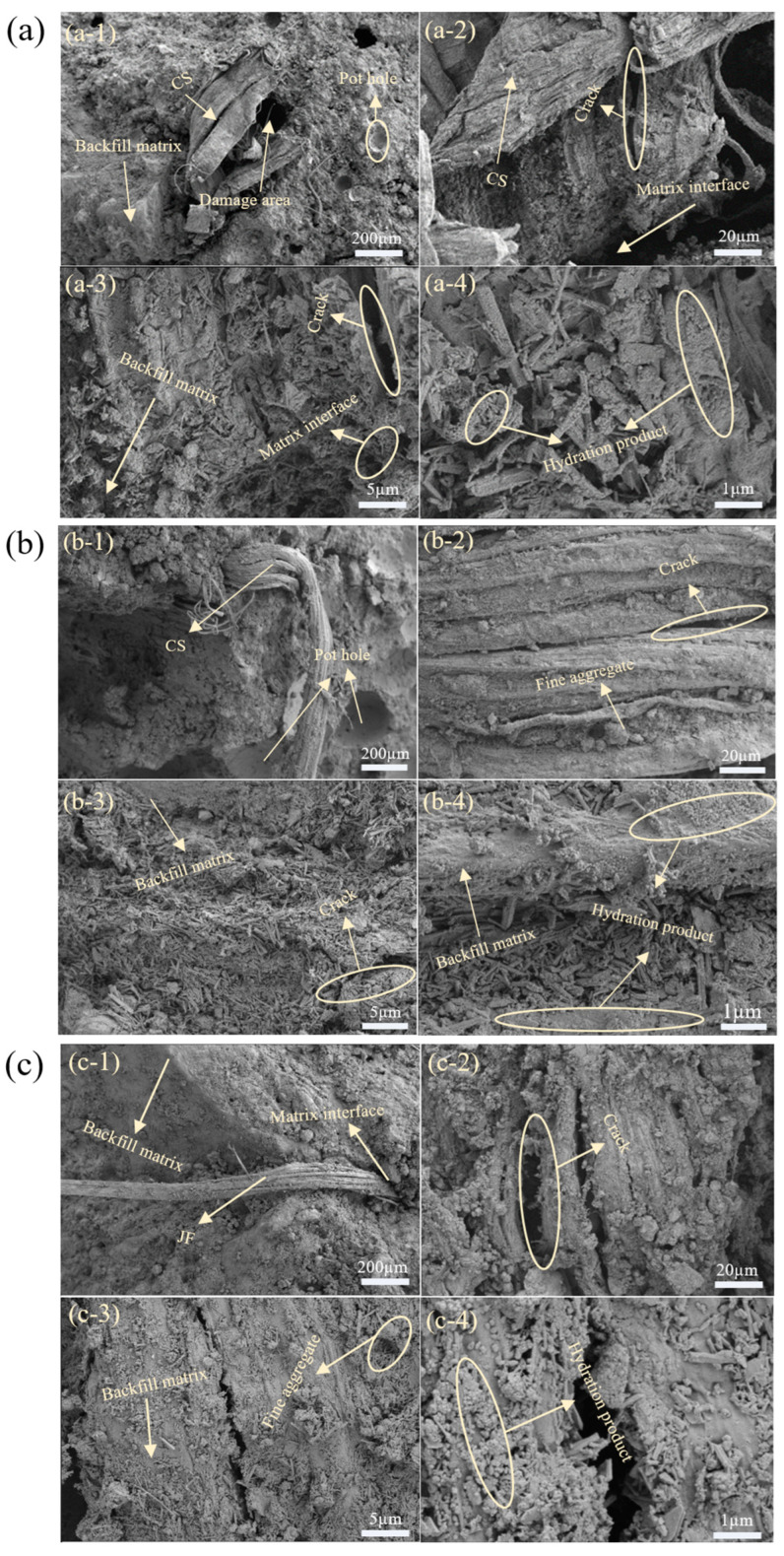
SEM images of samples with different natural fibres ((**a**) CS, (**b**) RS, (**c**) JF).

SEM analysis of the highest compressive strength samples at 28 days is shown in Figure 20, revealing micro-scale interactions between natural fibres and the backfill matrix. For CS, Figure 20(a-3,a-4) show fibre surfaces coated with cement hydration products, enhancing fibre-matrix bonding and enabling effective bridging. This bridging improves sample integrity, inhibits crack propagation, and, under load, fibre-induced tensile forces enhance mechanical strength [71].

**Figure 21:**
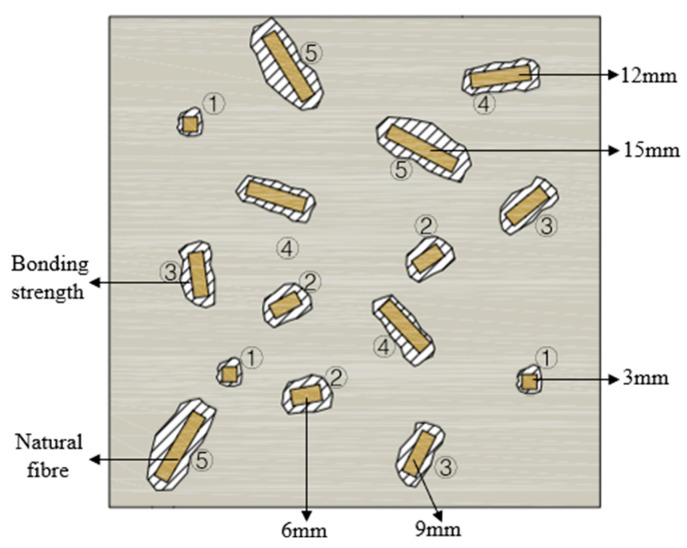
Bonding diagram of samples with different fibre lengths in backfill materials.

Figure 20(a-1,a-2) schematically illustrates fibres on a shear failure surface, where rough textures further strengthen bonding and bridging (Figure 21). However, cracks develop within fibres at high loads, reducing bonding and forming larger cracks. Beyond load thresholds, fibres deteriorate, weakening the bridge and initiating sample failure as fibres are pulled from the matrix [22,23,72]. RS and JF show similar failure trends (Figure 20b–c). As shown in Figure 20(b-3,b-4,c-3,c-4), the surfaces of both fibre types are only partially coated with hydration products. This coverage is relatively sparse, leading to weaker bonding with the matrix compared with CS. Moreover, Figure 20(b-1,b-2,c-1,c-2), show that RS and JF on the fracture surfaces have smaller contact areas and smoother surfaces than CS, which results in poorer interfacial bonding. Consequently, during matrix failure, these fibres are more readily pulled out.

Among the three fibres, CS-reinforced samples exhibit superior mechanical properties due to larger surface area and stronger bonding with the matrix [28,29,30]. In contrast, tubular RS is limited by length, and filamentous JF has a smaller surface area, explaining the differing effects on sample mechanical performance.

### 3.7. Environmental Impact Analysis

**Figure 22:**
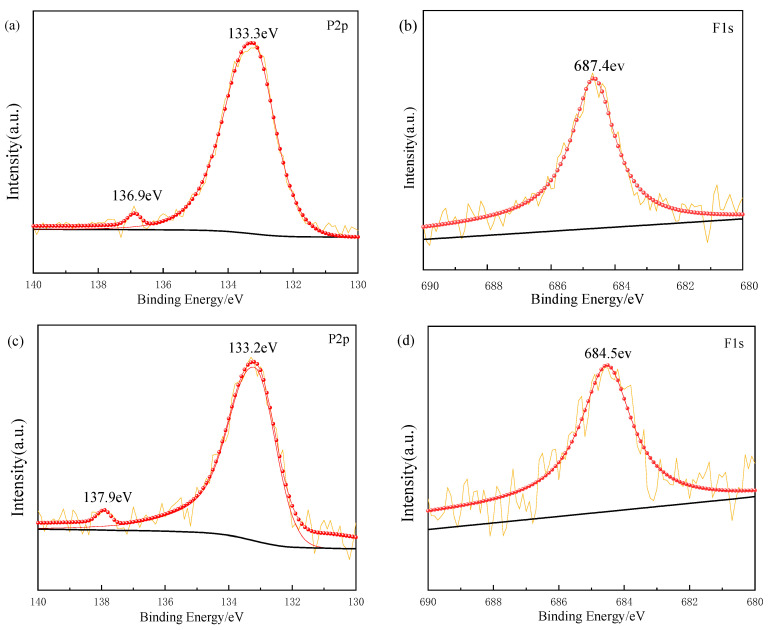
XPS spectra of the P2p and F1s orbitals for natural fibre samples and the Ref. ((**a**,**b**) CS; (**c**,**d**) Ref.).

Leaching test results and XPS analysis of the Ref. and CS-reinforced samples (12 mm, 0.2 wt%) are shown in Table 6 and Figure 22.

Incorporation of natural fibres significantly reduces P and F leaching, likely due to their large surface area and functional groups [73]. The XPS spectra reveal two characteristic P2p peaks. With fibre addition, one P2p peak shifts to lower binding energy, indicating an altered chemical environment, likely due to coordination bonding with oxygen in cellulose (e.g., P–O–C bonds). The increased P2p peak intensity in fibre-added samples further suggests enhanced adsorption or chemical binding of phosphorus.

For fluoride, immobilization occurs not only via precipitation with calcium oxide (CaO) but also through chemical interactions, as the F1s peak shifts to higher binding energy in fibre-incorporated samples, reflecting hydrogen bonding or surface complexation with fibre functional groups [74]. These mechanisms synergistically reduce fluoride mobility. Additionally, quantifying radionuclide content in phosphogypsum remains a key step for assessing environmental impact, warranting dedicated investigation in future studies.

## 4. Conclusions

This study evaluated the effects of three natural fibres on the mechanical properties and microstructure of backfill samples at varying fibre lengths and dosages. The main conclusions are as follows:

(1) Natural fibres reduce backfill fluidity and porosity, particularly 3 mm CS fibres at 0.2 wt%, while increasing pore density and resulting in a denser microstructure, as confirmed by NMR analysis.
(2) Compressive strength increases with fibre length (3–15 mm), with optimal lengths of 12 mm for CS and 3–9 mm for RS. Fibre content (0.1–0.5 wt%) showed an initial increase in compressive strength, followed by a decrease in CS and RS samples. JF samples exhibited an increase–decrease–increase pattern, with the greatest improvement for 0.2 wt% CS (12 mm), achieving compressive strength increases of 82.3%, 108.4%, and 144.4% at 7, 14, and 28 days, respectively.
(3) Tensile strength increased with fibre length (3–15 mm), with CS (9, 12, and 15 mm) and RS (3 mm) samples showing the most significant improvement. As fibre content increased (0.1–0.5 wt%), tensile strength in CS and RS initially rose, then declined, while JF samples showed a similar pattern. The greatest improvement was observed for CS at 0.4 wt% (12 mm), with an 18.8% increase in tensile strength compared to the reference.
(4) The addition of natural fibres changed failure modes from tensile-dominated (reference) to predominantly shear failure, improving sample integrity and fibre–matrix bonding. SEM and XRD analyses confirmed that fibre incorporation strengthened the matrix and provided bridging effects, reducing crack propagation. Ion leaching tests and XPS analysis revealed that fibres effectively adsorb and immobilize phosphorus (P) and fluorine (F), reducing the environmental impact of phosphate tailings.

## Figures and Tables

**Table 1:** The chemical composition of flotation and gravity tailings.

Types (%)	CaO	MgO	SO_3_	P_2_O_5_	SiO_2_	Al_2_O_3_	Fe_2_O_3_	K_2_O	TiO_2_
Flotation tailings	61.04	20.56	1.06	6.59	6.40	2.07	1.23	0.68	0.11
Gravity tailings	38.76	9.47	2.82	12.97	25.04	3.50	3.82	2.23	0.67

**Table 2:** Chemical composition of binding materials.

Types (%)	SO_3_	CaO	SiO_2_	Al_2_O_3_	Fe_2_O_3_	P_2_O_5_	K_2_O	MgO	TiO_2_
Phosphogypsum	43.09	38.29	12.64	1.66	1.05	0.99	0.92	0.77	0.47
Slag powder	2.10	44.40	27.85	14.46	0.20	0.33	0.35	7.92	1.53
Cement	0	65.90	22.27	5.59	3.47	0.07	0.70	0.81	0.31

**Table 3:** Chemical compositions of natural fibres.

Types (%)	Cellulose	Hemicellulose	Lignin
CS	21.98~28.40	25.12~27.87	14.27~15.82
RS	24.91~52.26	8.39~32.74	10.40~33.36
JF	55.36~65.58	19.46~25.50	8.68~13.37

**Table 4:** Mix proportions of samples.

Mix No.	Ref.	RS1~5	CS1~5	JF1~5	RS6~9	CS6~9	JF6~9
Solid content (wt%)	76 wt%						
Natural fibre length (mm)	0	3, 6, 9, 12, 15			12	3	12
Natural fibre content (wt%)	0	0.1			0.2, 0.3, 0.4, 0.5		
Research item	0	Fibre length effect			Fibre content effect		

**Table 5:** Comparison of effects of natural fibres on mechanical properties of backfill materials.

Fibre Type	Backfill Material	Compressive Strength (MPa)	Amplify (%)	Tensile Strength (MPa)	Amplify (%)	Reference
Corn straw	Phosphorus tailings	7.16	144.4%	1.20	18.8%	This study
Rice straw		4.93	68.3%	1.13	11.9%	
Jute		6.10	108.2%	1.16	14.9%	
Corn straw	Coal gangue	5.22	10.6%	0.85	14.8%	[20]
Polypropylene	Coal gangue	5.24	63.8%	——	——	[63]
Rice straw	Copper mine tailings	6.43	16.9%	1.05	31.2%	[66]
Rice straw	Lead and zinc mine tailings	3.38	19%	——	——	[25]
Polypropylene	Gold mine tailings	3.43	19.9%	——	——	[33]
Polyacrylonitrile		3.31	25.4%	——	——	
Glass		3.02	14.4%	——	——	
Polypropylene	Copper tailings	4.87	55.6%	0.33	21.7%	[67]

**Table 6:** Ion Test Results.

	P (mg/L)	F (mg/L)
Ref.	0.198	0.83
CS	0.063	0.67

## Data Availability

The original contributions presented in this study are included in the article. Further inquiries can be directed to the corresponding author.
